# Optical coherence tomography angiography as non-invasive tool for detection of characteristics patterns in circumscribed choroidal hemangioma

**DOI:** 10.1186/s40942-026-00864-9

**Published:** 2026-05-07

**Authors:** T. Kurdiani, M. Lever, K. Al-Ghazzawi, N. E. Bechrakis, T. Kiefer

**Affiliations:** Department of Ophthalmology, University Duisburg-Essen, University Hospital Essen, Hufelandstraße 55, 45147 Essen, Germany

## Abstract

**Introduction:**

Circumscribed choroidal hemangioma (CH) is a benign vascular intraocular tumor, most commonly diagnosed in adulthood. Clinical symptoms include visual disturbances caused by subretinal fluid and degenerative retinal changes involving the macula. Diagnosis is based on clinical examination; however, multimodal imaging techniques such as optical coherence tomography (OCT), fluorescein angiography (FA), indocyanine green angiography (ICGA), and ultrasound (US) are essential in differentiating from other intraocular tumor entities. While FA and ICGA are dye-related invasive methods with possible side effects, OCT Angiography (OCTA) is a non-invasive diagnostic method for visualizing retinal and choroidal vasculature. We aimed to explore OCTA as a noninvasive diagnostic modality for CH focusing on characteristical patterns.

**Methods:**

We conducted a retrospective analysis of 26 patients with untreated CH who underwent OCTA, as well as conventional FA and ICGA. OCTA images were evaluated for specific irregular vascular patterns within the choroidal vasculature, such as ‘bag of worms’, spaghetti-like/giant vessels, club-like appearance vessels with terminal bulbs, and avascular zones.

**Results:**

Of the 26 Patients analysed, 85% (22 eyes) exhibited irregular vascular patterns in the choroidal vasculature, as described above, and were labelled OCTA pattern present. Four eyes showed no such patterns and were labelled as OCTA pattern non-present. Compared to the second group, the OCTA pattern present group had significantly smaller tumor thickness (1.87 vs. 3.53 mm; *p* = 0.0001).

**Conclusion:**

These findings suggest that OCTA can effectively detect characteristic changes in CH, particularly in lesions with tumor prominence below 3 mm. OCTA has the potential to become a valuable non-invasive tool for CH diagnosis; however, larger studies are needed to further validate its clinical utility.

**Supplementary Information:**

The online version contains supplementary material available at 10.1186/s40942-026-00864-9.

## Introduction

Circumscribed choroidal hemangioma (CH) is a benign vascular tumor of the choroid, characterized by solitary lesions with well-defined borders, typically located in the posterior pole or near the vascular arcades beyond the equator [1]. CH is believed to be a congenital disease; However, most patients tend to develop symptoms and seek medical help in their adulthood, typically during their fourth to sixth decades of life [2]. This condition is sporadic and exhibits a slight male predominance [3–6]. Furthermore, CH appears to be more prevalent among individuals of Caucasian descent [1, 7].

The precise incidence of CH is uncertain, as it is usually identified only in symptomatic cases or incidentally. Nevertheless, it is estimated that there is approximately one case of CH for every 15 cases of choroidal melanoma [1].

Most CH patients are diagnosed due to visual symptoms caused by the accumulation of serous subretinal fluid, degenerative macular alterations, or a combination of these factors. This reddish-orange, circular to oval choroidal tumor is typically located mainly or entirely posterior to the equator. The posterior border of most CH are situated within two-disc diameters from the optic disc, foveola, or both. The retinal pigment epithelium overlying the lesion often undergoes degenerative alterations, which may include fibrous metaplasia and, occasionally, degenerative calcification and even ossification. Serous retinal detachment is a common complication and can sometimes be misdiagnosed as central serous chorioretinopathy [8].

The diagnosis of CH is based on its typical clinical appearance combined with multimodal imaging techniques, including ultrasonography, optical coherence tomography (OCT), and fluorescein and indocyanine green angiography (FA & ICGA). FA typically shows the early hyperfluorescence of larger choroidal blood vessels, either preceding or occurring simultaneously with the initial filling of retinal arterioles. As imaging progresses, fluorescein often stains the entire lesion and any accompanying subretinal fluid. ICGA demonstrates the filling of intralesional vascular channels and exibits intense hypercyanescence of the lesion in the intermediate frames, peaking around 3–4 min, followed by a gradual washout of the lesion’s central portion in the late-phase. This imaging technique typically provides a much clearer view of the full extent of a CH [8, 9]. Nevertheless, FA and ICGA are invasive diagnostic procedures that potentially are associated with side effects such as nausea or allergic reactions up to allergic shock, eventough rare [10].

OCT angiography (OCTA) is a non-invasive imaging technique used to visualize retinal and choroidal vessels. In clinical practice, it offers a promising tool for assessing and potentially guiding the treatment of various retinal pathologies such as diabetic retinopathy or choroidal neovascularization in age-related macular degeneration [11, 12].

OCTA has rarely been used for diagnosis of CH with just a few reports in the literature, however there exist evidence that typical vasculature patterns can be observed in the choroid using OCTA [10, 13, 14]. So far, these findings have been difficult to validate in a larger patient cohort. Additionaly distribution of vascular patterns in the CH spectrum and possibly disadvantages in thicker tumors possibly inhibiting visualization of these characteristics have barely been investigated in the existing literature. We therefore conducted this study to add further evidence in the use of OCTA for the diagnosis of CH, analyzing choroidal vasculature and specifying diagnostically helpful patterns.

## Methods

A retrospective case analysis was conducted on all patients with CH examined at the Department of Ophthalmology of the University Hospital Essen between October 2022 and September 2023. CH patients were analyzed with the following inclusion criteria: OCTA images available, treatment-naïve tumors and tumors in the foveol region, juxtapapilläre or within or close to the vascular arcades. OCTA was obtained with SPECTRALIS^®^ HRA + OCT device (Heidelberg Engineering; Heidelberg, Germany) using a 15°x15° Field of View. Patients with incomplete clinical, uncertainty in clinical diagnosis, pretreated eyes, comorbitities that could possibly influence (choroidal slab) OCTA analysis (e.g. pachychoroid spectrum disease, chorioretinitis, choroidal neovascularization) as well as poor image quality, were excluded from the study. Poor image quality was defined by one or more of the following criteria: decentered image with insufficient area of tumor choroid represented, presence artifacts which altered choroidal slab analysis & poor image quality due to alteration of optical media (e.g. cataract, corneal opacities). Following clinical data were collected: age, sex, and corrected distance visual acuity (CDVA), tumor thickness, tumor maximal and minimal diameter by ultrasound (Eye cubed from Ellex, Berlin, Germany). Additional multimodal diagnostic imaging included widefield fundus photography (ZEISS Clarus 700, Jena, Germany), macular OCT, FA, and ICG if applicable (SPECTRALIS HRA + OCT, Heidelberg, Engineering; Heidelberg, Germany).

OCTA images were analyzed for the presence of vascular patterns in the choroidal slab. These patterns were the following: bag of worms (area of packed small curved vessels) spaghetti/giant vessels (large caliber bent vessels); club-like vasculature (vessels with small base and thicker terminus), vessels with terminal bulbs (vessels with bulb like branches and endings) and avascular zones (area with lack of vessels or vessel signal respectively). For OCTA Imaging analysis, automatic segmentation was used to delineate choroidal layers which included as much choroidal /tumor vasculature as possible, provided by the instrument manufacturer (software HEYEX Version 2.5.5 In cases where automated segmentation was inaccurate, manual correction was performed by the readers. Choriocapillaris slab was excluded as good as possible as it is usually preserved in CH as compared to other choroidal tumor entities [15]. OCTA images were pseudonymized for image analysis.

OCTA patterns were analyzed and determined by two experienced clinical ophthalmologist (reader 1: resident, reader 2: consultant) in consensus masked to clinical data and other image modalities.

The study protocol was reviewed and approved by the local ethics committee (University of Duisburg Essen) under approval number 23-11553-BO in accordance with the Declaration of Helsinki. Patients gave informed consent.

### Statistical methods

Data were collected in Microsoft Excel (Microsoft, Redmond, WA, USA). Normal distribution was assessed with the D’Agostino and Pearson normality test. Mean values of continuous data were compared with the non-parametric Mann-Whitney U test, when appropriate. Receiver Operation Curve (ROC) Analysis was performed to analyze possible cut-off value of tumor thickness for lack of detection of OCTA characteristics. Statistical analyses were performed using Prism 10.1.3 (GraphPad, La Jolla, CA, USA). Dichotomous variables are presented as absolute and relative frequencies (n, %) and continuous variables as mean ± standard deviation (SD). Statistical significance was asserted for p-values < 0.05.

## Results

### Clinical presentation

We included 26 eyes of 26 patients with untreated CH who underwent OCTA imaging in our clinic. Clinical characteristics are summarized in Table 1. The mean age at the time of visit was 57 years (range, 29–77 years). Ten patients were female, and sixteen were male. Mean corrected distance visual acuity (CDVA) was 0.63 logMAR (range, 1.7-0 logMAR). The mean tumor thickness was 2.1 mm (range, 1.0–3.7 mm). Mean basal diameter was 6.8 mm (range, 3.98–10.8 mm) for minimal and 8.1 mm (range, 4.6–11.7 mm) for maximal diameter, respectively.

**Table 1:** Clinical and imaging characteristics of 26 non-treated choroidal hemangioma patients

	Mean (*n* = 26 10female:16male)	Mean standard deviation	Range
Age	57	± 10.6	29–77
CDVA (Log Mar)	0.63	± 0.52	1.7–0
Tumor thickness (mm) *	2.1	± 0.77	1.0 − 3.7
Basal diameter min (mm) *	6.8	± 1.65	3.98–10.8
Basal diameter max (mm) *	8.1	± 1.82	4.6–11.7
Histological Validation	2 (7.6%)	-	
FA	25 (96%)	-	
ICG	12 (46%)	-	
Subretinal fluid on Tumor ^+^	25 (96%)	-	
Foveal subretinal fluid ^+^	19 (73%)	-	
Intraretinal fluid ^+^	18 (69%	-	
Pigment epithelium atrophy ^+^	18 (69%)	-	
Subretinal fibrosis ^+^	7 (27%)	-	

CDVA = corrected distance visual acuity; OCTA = optical coherence tomography angiography. Values are presented as mean ± standard deviation unless otherwise stated. Min=minimal. Max=maximal. FA: fluorescein angiograohy. ICG: indocyaningreen angiograohy. *: measured by ultrasonography, *: determined by OCT

All 26 patients underwent B–scan ultrasonography, which demonstrated a characteristic hyperreflective or isoreflective choroidal mass in all cases (100%).

OCT was performed on all 26 eyes for the diagnosis of CH, with 25 eyes showing subretinal fluid over the choroidal lesion (96%). In 19 of these eyes, the subretinal fluid involved the fovea (73%). Subretinal fluid was determined in all cases by OCT. Additionally, intraretinal fluid was presend in 18 tumors (69%), pigment epithelium atrophy in 18 tumors (69%) and subretinal fibrosis in 7 tumors (27%).

Fluorescein angiography (FA) was performed in 25 eyes (96%) and demonstrated the characteristic angiographic features previously described. Indocyanine green angiography (ICG) was conducted in 12 eyes (46%), all of which exhibited the typical presentation as previously reported.

**Fig. 1:**
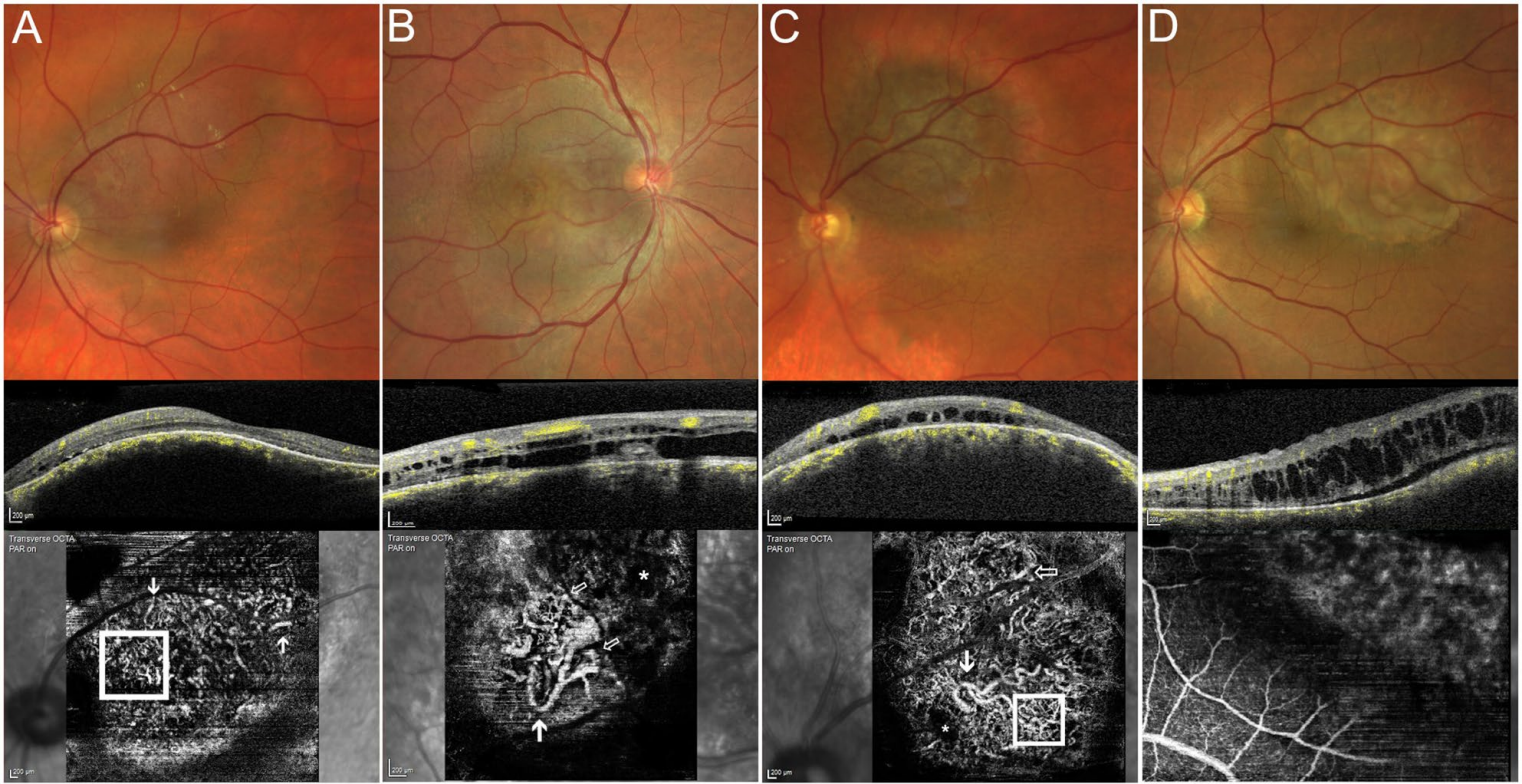
Four examples of choroidal OCT Angiography in choroidal hemangioma: **A**: macular hemangioma with present spaghetti/giant-vessels (thick arrow), terminal light bulbs (light arrows) and avascular zone (*). **B**: juxtapapillary hemangioma with present bag of worms (square) and club-like vasculature (arrows). **C**: juxtapapillar hemangioma with present spaghetti/giant vessels (thick arrow), club-like vasculature (light arrow), bag of worms (square) and avascular zone (*). **D**: 3.7 mm prominent hemangioma located at posterior pole with insufficient depiction of choroidal vasculature patterns

The diagnosis of CH was established based on clinical and multimodal imaging findings in 24 eyes. However, two eyes underwent transretinal biopsy via pars plana vitrectomy due to uncertainties in clinical diagnosis in tumors with tumor thickness of more than 3 mm. One Case was accompanied by high amount of inferior serous retinal detachment with uncertainty in differential diagnosis to choroidal metastasis and another case showed some degree of orange-pigemt with uncertainty in differential diagnosis to choroidal melanoma (Fig. 1D). Histopathology in these cases revealed typical vascular malformations of the choroid consistent with CH. Immunohistochemistry showed positive staining for CD31 and CD 34 (vascular endothelial markers) [16]. Molecular genetics showed a mutation of the GNAQ 209 Gene in one patient, consistent with CH [17], whereas no GNAQ 209 mutation was detected in the second patient.

### Optical coherence tomography angiography

Irregular vascular patterns within the choroidal vasculature were observed on OCTA in 22 of 26 eyes. These included a “bag of worms” appearance, spaghetti-like vessels, club-shaped vasculature, vessels with terminal bulb dilatations, and avascular zones, all of which are considered characteristic of CH. These patterns were consistent with the diagnosis of CH. Representative examples are shown in Fig. 1. Such changes were visible in either the choriocapillaris or deeper choroidal layers. For the purposes of this analysis, these patients were classified as OCTA Positive.

The distribution of vascular patterns observed in the study cohort was as follows: Choroidal vasculature exhibiting a “bag of worms” morphology was identified in 14 of 22 eyes (63.6%). A “spaghetti-like” pattern was observed in 10 eyes (45.4%), while a “club-like” vascular configuration was present in 18 eyes (81.8%). Vessels with terminal bulb formations were detected in 21 eyes (95.4%), and avascular zones were noted in 10 eyes (45.4%). A detailed summary of these findings is provided in Table 2.

**Table 2:** Distribution of vascular patterns in OCTA pattern present eyes analyzing choroidal vasculature

Vascular Pattern	Number of Eyes (*n* = 22)	Percentage (%)
“Bag of worms” morphology	14	63.6
“Spaghetti-like” pattern	10	45.4
“Club-like” vascular configuration	18	81.8
Terminal bulb formations	21	95.4
Avascular zones	10	45.4

In 4 out of 26 eyes, no clearly irregularly arranged choroidal vessels (as defined above) could be identified. These eyes were therefore classified as OCTA patterns presence (OCTA+). OCTA images in these cases showed artefacts, resulting in insufficient visualization and interpretability of the choroidal vasculature. This means that even though some kind of typical choroidal patterns were perceivable, they were not clearly distinguishable to be classified as OCTA patterns non-present (OCTA-).

When comparing OCTA + to OCTA- eyes, tumors with present patterns eyes had significantly lower mean tumor thickness (1.9 mm vs. 3.5 mm; *p* = 0.0001) as well as minimal and maximal basal tumor diameter (6.3 mm vs. 9.2 mm; 7.7 mm vs. 10.1 mm; *p* = 0.0001 & 0.01 respectively). ROC analysis showed tumor thickness of less than 3 mm had perfect sensitivy and specificity (100%) and maximal diameter of less than 9,7 mm had best sensitivity (75%) and specificity (86%) in our cohort for presence of OCTA characteristics. Clinical observations are shown in Table 3.

**Table 3:** Shows clinical data differences between OCTA pattern present vs. OCTA pattern non-present in non-treated eyes

	OCTA patterns present (*n* = 22)	OCTA patterns non-present (*n* = 4)	*P* value
CDVA LogMAR	0.62	0.67	0.97
Mean tumor thickness(mm)	1.87	3.53	< 0.0001
Mean minimal diameter mean(mm)	6.3	9.15	< 0.0001
Mean maximal diameter(mm)	7.74	10.08	0.011
Subretinal fluid	21 (95%)	4 (100%)	0.65
Intraretinal fluid	15 (68%)	3 (75%)	0.78
Pigment epithelium atrophy	16 (73%)	2 (50%)	0.37
Subretinal fibrosis	6 (27%)	1 (25%)	0.94

CDVA = corrected distance visual acuity; OCTA = optical coherence tomography angiography. *P* values were calculated using appropriate statistical tests

Notably, the two eyes that underwent transretinal biopsy, as described above, were classified as OCT-. In both cases, the OCTA image quality was inadequate due to pronounced artifacts, preventing the identification and characterization of any vascular patterns.

### Comparison to ICG

**Fig. 2:**
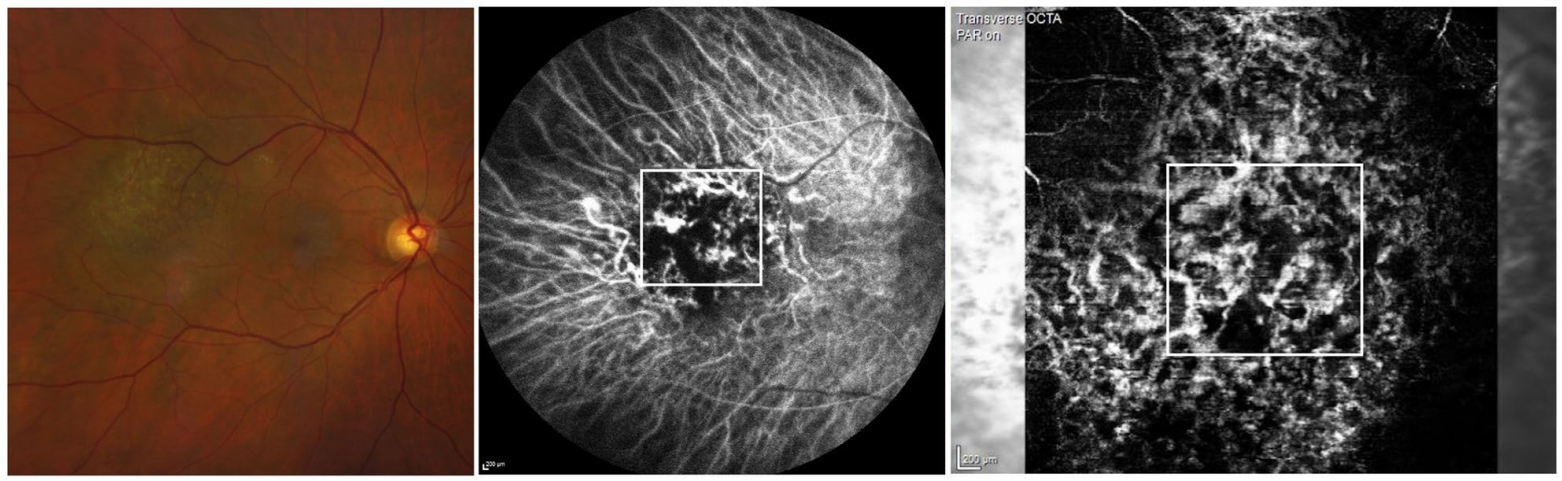
Example of circumscribed choroidal haemangioma comparing early phase Indiocyaningreen-angiography with OCT Angiography. The corresponding areas are depicted in the white marked area. OCTA presents club-like vasculature, bag of worms and terminal light bulbs with only the latter two redistinguable in the Indiocyaningreen-angiography

9 out of 22 OCTA+ eyes also had ICG images available. All ICG images showed intrinsic tumor vasculature in the very early phase, that were later indistinguishable due to intense hypercyanescence after a few minutes, and were no longer detectable in the washout phase. Interestingly, all intrinsic tumor vasculature could be similar displayable in the corresponding OCTA pictures to some extend (Fig. 2). We tried to compare presence of OCTA patterns per patient as described above with early phase ICG images retrospectively non-blinded. Following that we could find full agreement in agreement in 3 cases (33%) and partial agreement in 6 cases (66%). OCTA pattern that was most common found in OCTA and redetected in ICG were terminal bulbs in 6 cases, followed by giant vessels (4 cases), club-like pattern (4 cases) and bag of worms (3 cases). Visible OCTA pattern that were not be depictable in early phase ICG were club-like pattern (3 cases), bag of worms (3 cases) and avascular zone (2 cases).

Furthermore, 3 out of 4 OCTA-tumor could show intrinsic tumor vasculature in the early phase of ICG which could not be imagined by OCTA as described above.

## Discussion

Our study demonstrated that OCTA can detect characteristic choroidal vascular patterns of CH in the majority of untreated eyes, with positive findings in 85% of the whole cohort and in all tumors with less than 3 mm tumor thickness.

OCTA is a non-invasive diagnostic imaging technique for visualizing retinal and choroidal vessels [18]. It works by detecting the interference between light reflected from a reference mirror and light scattered back from a biological sample, enabling the measurement of backscattered light intensity at different depths within the tissue. Compared with FA and/or ICG, OCTA offers the advantage of visualizing vascularization without the need to inject any dye, which can potentially lead to allergic reactions up to allergic shock and less threatening but more common side effects inclusidng cills, hot flushes and inflammation at the injection site. Even though these complications are rare, FA should not be performed routinely in every patient visit, due to its invasive nature [19]. This is when OCTA can be valuable, as it avoids the aforementioned complications and enables repeated examinations and sequential comparisons.

Currently, there is limited literature on the use of OCTA for diagnosing and visualization of CH. To our knowledge this is largest cohort of OCTA analysis in CH, particullary with focus on potential characteristic OCTA pattern. Additionally, from our best awareness there has been no other investigation of OCTA pattern distribution and comparison with ICG. Majority of existing publications tend to consist either of case reports or of small patient cohorts. Most of former literature describe choroidal vessel irregularities that align with our observations. For example, choroidal vessel irregularities have been described as ‘richly branched and voluminous vascular pattern and wrapped by rarefied vessels peripherally’ [20] or ‘dense irregular vascular network in the choroid capillary layers’ [21]. Gündüz et al. were able to detect internal tumor vessels described as ‘multiple dilated interconnected club-shaped tumor vessels’ in all of their relative big cohort of 20 tumors which aligns to our findings [22]. Additionally, they interpreted signal void areas corresponding to avascular areas in our study as ‘intervening connective tissue’ with can be find in a subcohort of capillary CH in histopathological studies [15]. Another relatively large cohort of Kreminger et al. including 19 patients concentrated on quantitative measurements such as choroid vascularity index using Swept-Source OCTA [23]. In accordance with our findings, intra- oder subretinal fluids did not alter OCTA vascular analysis in tumor eyes. However, they were not able to detect spefic OCTA patterns as opposed to our findings and other literature in their study. This could possibly explained by concentration of choriocapillaris slab in contrast to our strategy of analysis of deeper choroideal vasculature as choriocapillaris is usually preserved in CH in contrast to other choroidal tumor entities [15]. Altough we aimed to define clear OCTA patterns and tried to analyse it distribution in a larger cohort, there must not be neglected that no consensus in definition and nomenclature of different OCTA patterns for CH exists, reflected by vary of terminology used in the literature.

Our study does not include pre-treated eyes; however, in our clinical practice, we have observed no consistent vascular patterns in such patients. Opposed to that different small case series tried to elucidate OCTA changes after therapy. For example Chawla et al. demonstrated absence of choriocapillaris after laser photocoagulation, but their cases demonstrated choroid vasculature in OCTA scans in aligmement with our findings [24]. Mirzayev et al. demonstrated decrease in tumor vascularity with increase of flow void / avascular area possibly explained by increase of fibrous components after transpupillary thermotherapy [25].

Although OCTA appears to be a promising diagnostic tool for CH, it has several limitations. In our study population, tumors greater than 3 mm exhibit artifacts that may hinder visualization of vascular structures within the tumor. We strongly believe lack of imagination of the described characteristics in tumor greater than 3 mm does not imply lack of vascular patterns per se but difficulties in visualization due to altered image quality including lack of correct segmetation, signal attenuation and projection artifacts. This most possibly results by tumor volume not by other structural changes such as subretinal fluid, pigment epithelium alterations or subretinal fibrosis as showed in our cohort. Possibly these limitations of OCT in more prominent tumors – not only in CH but other differential diagnosis in general – can be overcome by future improvements in OCTA technology. As a result, other diagnostic methods, such as ultrasonography, FA, ICGA, or possibly histological validation, still serve as gold standard and we rather see OCTA as additional tool in the toolbox of multimodal CH imaging. As such its diagnostic ability to replace invasive dye-based angiography modalities has not been yet sufficiently verified. However, there might be a clinical role when such angiography settings are not feasible – for example in incompliant or phobic patients – or not obtainable. Compared to ICG it bring the potential benefit of time-independence in visualization of intrinsic tumor vessels, has lesions appear uniform hypercyanescent in the later ICG phase. Additionally, we could show that OCT patterns could be recognizable in early ICG phase, however there was only partial consistency in the majority of cases where both modalities were available and this analysis was not blinded by OCTA results.

The limitations of our study are the retrospective design and the lack of a control group. Therefore, it would be desirable to compare OCTA imaging with other choroidal tumor entities, such as (amelanotic) choroidal nevi or melanoma, which are important differential diagnoses to CH. Altough we believe our findings represent specific findings for CH by our knowledge of analysis in other choroideal tumor entities [26], our study is not suitable for definitely answer that question and further research comparing its ability in diagnosis CH by OCT in comparison to other differential diagnosos. Furthermore, our cohort was to small and imbalanced to overemphasize statistical results and proposed cut-off values for tumor thickness in our study. Determination of OCT patterns may possibly be reader dependent and prone to subjectivity. To address this, definition of each pattern as described above was made before image analysis and classification was made by two readers in consensus. Revalidation of these results should be obtained in future studies with independent reader to study consistency of OCTA pattern. Additionally, quantitative measurements of choroidal vasculature or blood flow that are not dependent on potentially subjective reader interpretation would be helpful in objectifying the evaluation of OCTA images in CH patients. Currently, many commercially available OCTA software lacks as in our study such quantitative capabilities, limiting reproducibility and standardization of these evaluations. Unfortunately, we needed to exclude peripheral CH in our study due to poor image quality. However, the majority of CH cases are located in the posterior pole and close to the vascular arcades. As such we still believe our results represent a reasonable cohort to general CH population.

## Conclusion

Our results demonstrate that OCTA is feasible to detect characteristic changes in CH, particularly in lesions with a tumor thickness of less than 3 mm. While eyes with a lower tumor thickness consistently showed typical OCTA changes in the choroidal vasculature -such as “bag of worms”, “spaghetti like vessels “, and vessels with terminal bulbs-these changes were not depictable in eyes with thicker tumors, which we particulary see as technical limitation in more prominent tumors but not as specific finding in larger CH.

In conclusion, OCTA has the potential to become an additional non-invasive method extending the toolkit of multimodal imaging in assessing lesions of the posterior pole, such as CH. As OCTA is non-invasive and poses no health risks to patients. Larger studies with matched control groups are needed to further validate our results, prove potential independent reproducibility and emphasize the usefulness of OCTA in the diagnosis of neoplasms of the posterior pole, such as CH.

## Electronic Supplementary Material

Below is the link to the electronic supplementary material.

Supplementary Material 1

## Data Availability

The dataset supporting the conclusions of this article is included within the article as additional/supplemental file.

## Abbreviations

CH: Circumscribed choroidal hemangioma
OCT: Optical coherence tomography
FA: Fluorescein angiography
US: Indocyanine green angiography (ICGA) ultrasound
OCTA: OCT Angiography
CDVA: Corrected distance visual acuity
SD: Standard deviation
